# Uterine Development During Induced Puberty in Girls with Turner Syndrome

**DOI:** 10.3389/fendo.2021.707031

**Published:** 2021-07-06

**Authors:** Monika Obara-Moszynska, Lukasz Dzialach, Barbara Rabska-Pietrzak, Marek Niedziela, Karina Kapczuk

**Affiliations:** ^1^ Department of Paediatric Endocrinology and Rheumatology, Institute of Pediatrics, Poznan University of Medical Sciences, Poznan, Poland; ^2^ Student Scientific Society of Paediatric Endocrinology, Poznan University of Medical Sciences, Poznan, Poland; ^3^ Department of Gynaecology, Poznan University of Medical Sciences, Poznan, Poland

**Keywords:** Turner syndrome, puberty induction, uterine development, uterine volume, estrogen therapy

## Abstract

**Objective:**

Most girls and women with Turner syndrome (TS) require estrogen replacement therapy (ERT) to initiate or maintain pubertal development. Most likely, the most fundamental effect of ERT in hypogonadism is the promotion of uterine growth. The optimal ERT model is still being discussed. The present study aimed to assess uterine size in girls with TS in the prepubertal state during and after the induction of puberty and compare it to a healthy population.

**Methods:**

The analysis encompassed 40 TS girls. The prepubertal and postpubertal control groups contained 20 healthy girls each. All patients with TS were treated with 17-ß estradiol. Uterine imaging was performed with two-dimensional (2D) transabdominal ultrasound. The uterine volume (UV) and fundocervical antero-posterior ratio (FCR) were calculated in patients with TS before the pubertal induction, after 6-12 months of estrogen replacement therapy (ERT), after ≥ 36 months of ERT or ≥ 12 months after menarche.

**Results:**

The average age of TS patients at estrogen introduction and at the last control visit, when the uterus was considered mature, was 12.9 years and 16.1 years, respectively. The UV in patients with TS at the beginning of ERT was 1.55 ± 1.22 cm^3^ and was not significantly different from the UV in the prepubertal controls. The mature UV in patients with TS was 31.04 ± 11.78 cm^3^ and was significantly smaller than the UV of the postpubertal controls (45.68 ± 12.51 cm^3^, p<0.001). The FCR in girls with TS did not differ significantly from that in the prepubertal and postpubertal control groups, respectively. No prognostic factors could be established for the final UV. By the last control visit, thelarche had advanced in most patients to Tanner 4 and 5 (37.5% and 40%, respectively).

**Conclusions:**

Before the onset of ERT, patients with TS have a uterus similar in size to that in prepubertal healthy girls. Pubertal induction in patients with TS causes a significant increase in the UV that is detectable after 6-12 months of ERT. The mature uterus is smaller in patients with TS than in the age-matched healthy population.

## Introduction

Turner syndrome (TS) is a sex chromosome abnormality. TS is caused by complete or partial loss of one of the X chromosomes in a phenotypic female. The phenotypical spectrum is wide, but some of the features are classic in TS, particularly short stature, ovarian dysgenesis, infertility, and cardiovascular malformations (1). Although some girls with TS present spontaneous puberty, approximately 90% of girls and women with TS require or will require estrogen replacement therapy (ERT) to initiate or maintain pubertal development (2). It is recommended that women with TS receive estrogen and progestin replacement, which allows them to achieve pubertal features and to subsequently maintain secondary sex characteristics, attain peak bone mass and normalize uterine growth for possible future pregnancy (3). Most likely, the promotion of uterine growth is one of the most fundamental effects of estrogen therapy in hypogonadism (4). Adequate uterine development is a substantial determinant of positive pregnancy outcomes. TS patients with ovarian dysgenesis will not be able to become pregnant with their own eggs, but they may consider oocyte donation to achieve pregnancy, especially when the estimated risk of aortic dissection or aortic rupture is low due to the lack of identifiable risk factors (3). Uterine development takes several years, exhibiting growth from a small tubular organ to a mature heart-shaped structure throughout development (4). Some uterine growth is noticeable at the age of 6 years, but the majority occurs during puberty and continues beyond the time of menarche and full breast development (5, 6). Lack of estrogens leads to hypoplasia of the uterus (7, 8).

Estrogen replacement also has an important role in the growth process, metabolism, and psychological functioning (9, 10). The guidelines from 2017 recommend that ERT in TS should start at an age between 11 and 12 years, suggest that estrogen be administered in the form of estradiol (E2), and advocate a preference for the transdermal (TD) route (3). The optimal ERT regimen to induce pubertal development is still being evaluated. There is no study to date of TD administration from the initiation of puberty until adulthood (3). The TD method is suggested because it is more physiologic than the oral route, is not associated with the first pass through the liver and is not linked to a pro-coagulable state. Estrogen therapy aims to mimic physiology and not impair final growth. Delaying estrogen replacement may be deleterious to bone and uterine health, as observed in other forms of absent puberty (3). Objective uterine measurements in TS patients may be used to monitor estrogen therapy. In a modern strategy of pubertal induction in girls with TS, proposed by Donaldson et al. (11), uterine assessment with pelvic ultrasound is among the recommended measurements to be taken before, during, and at the end of the treatment. Data concerning the influence of different routes of estrogen therapy on uterine volume (UV) are still not clear because the route, dose, age at onset of treatment, and duration of the treatment may all influence uterine growth (3). Some studies have reported that UV is not affected by the type of estrogen used (12, 13) but is related to the dose and duration of therapy (14, 15).

The lack of universal definition for mature uterine dimensions and common agreed measurements for uterine size indicating maturity are additional problems in estimating uterus development in TS.

All these facts lead to many questions: What is the optimal kind of estrogen therapy, which can not only yield pubertal development but also induce a fully mature uterus comparable to that in healthy adult women? Is pubertal induction starting at an early age of 11-12 years able to guarantee a normal-sized uterus? Finally, is TS connected genetically with a small uterus, and what may determine the estrogen therapy response?

The present study aimed to assess uterine size in TS girls in the prepubertal state, during and after the induction of puberty, which started at the recommended age and was mostly transdermal, and compared this size to that in a healthy population. Furthermore, we tried to estimate the prognostic factors for uterine development, determining the normal adult uterine size.

## Materials and Methods

Forty patients with TS and hypergonadotropic hypogonadism, who were under the care of the Department of Paediatric Endocrinology and Rheumatology, Karol Jonscher’s Clinical Hospital of the Poznan University of Medical Sciences (PUMS), were analysed. The prepubertal control group consisted of 20 healthy prepubertal (Tanner 1) girls aged ≥ 5 and < 8 years (average age of 6.7 ± 1.0 years). The postpubertal control group consisted of 20 healthy adolescents with regular menstrual cycles who were ≥ 2 years after menarche (average age: 16.4 ± 2.0 years, gynaecological age: 4.5 ± 1.5 years). The prepubertal and postpubertal controls received a consultation in the Outpatient Clinic of Paediatric and Adolescent Gynaecology of Gynaecology and Obstetrics Clinical Hospital, PUMS, and were not diagnosed with any developmental or endocrine disorders, which may influence the uterine size.

Institutional Review Board approval was not obtained because the patients’ data were analysed retrospectively, and the performed tests were part of routine diagnostics. In accordance with our institution’s ethics guidelines, approval to conduct this study was not required (retrospective analysis of medical records). All patients and their parents were informed that the patients’ medical records might be used for research and scientific publications and signed informed consent forms.

Standard deviation (SD) for height and BMI percentiles were calculated using OLAF calculator according to normal Polish ranges (16). All TS patients were treated with 17-ß estradiol (E2): 35 girls (87.5%) used transdermal (TD) preparations (patches: Oesclim - Mylan, Estraderm MX – Merus Labs, or spray: Lenzetto – Gedeon Richter), and five girls (12.5%) received E2 orally (tablets: Estrofem mite – Novo Nordisk). The dose regimen (referring to transdermal therapy) is described below. During the first six months, 0.1 µg/kg was applied during the night; in the next six months, 0.1 was μg/kg applied continuously; in the second year of treatment, the E2 dose was increased to 2 µg/kg for the first 6 months and to 3 µg/kg for the next 6 months. Further dose adjustments were personalized according to the progress of pubertal features, growth rate, and serum E2 concentrations. The dose was adjusted every 6 months. The final dose ranged from 12.5 to 50 µg/d.

When calculating the dose of E2 in kg body weight, a 1 mg tablet was assumed to equal a 50 µg patch. When the first menstrual bleeding occurred or after two years of estrogen therapy alone, progestin (dydrogesterone) therapy was added (Duphaston – Mylan, 10 days monthly). Dydrogesterone is a progestin closely related to natural progesterone but it is bioavailable after oral administration. Therefore, we chose dydrogesterone to control endometrial proliferation.

Thirty-nine patients (97.5%) were treated with recombinant growth hormone (GH) (Omnitrope - Sandoz) at an average dose of 0.025 – 0.055 mg per kg body weight per day.

The control visits took place every six months.

**Figure 1:**
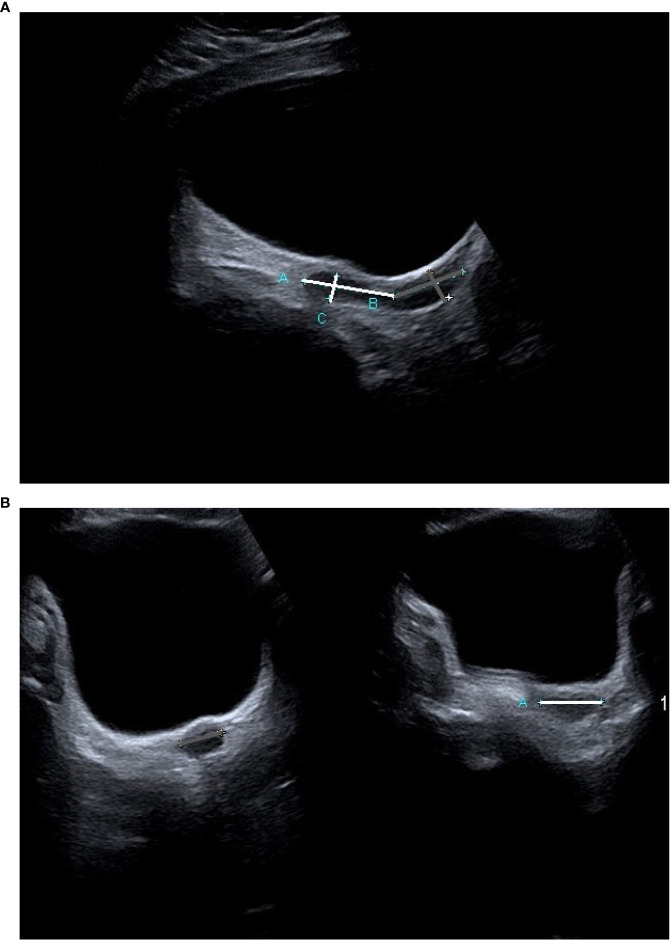
A transabdominal ultrasound image of the uterus in a TS patient prior to the beginning of ERT **(A)** longitudinal projection, **(B)** transverse projection). The measurements of the uterine body are marked white, and those of the cervix are marked grey.

Uterine imaging was performed with two-dimensional (2D) transabdominal ultrasound (TAUS) using an Aplio 400 Toshiba Premium, head 10C3. The same investigator (KK) performed all the examinations. In postmenarcheal girls, US was performed randomly in the menstrual cycle. Uterine length (L) (including length of the body (Lb) and of the cervix (Lc)), the antero-posterior diameter (AP) of the uterine body (APb) and of the cervix (APc), and the transverse diameter (T) of the uterine body (Tb) and of the cervix (Tc) were measured (**Figures 1A, B**). Endometrial thickness (E) was measured in the longitudinal scan. In girls with TS before the beginning of pubertal induction and in prepubertal controls, UV was calculated by assuming the form to be an elliptical cylinder using the volume formula: $\text{L}\times \frac{1}{2}\frac{\text{APb}+\text{APc}}{2}\times \frac{1}{2}\frac{\text{Tb}+\text{Tc}}{2}\times \pi$. In girls with TS after the beginning of pubertal induction and in postmenarcheal controls, UV was calculated by assuming the form of the uterine body to be ellipsoid using the volume formula: $\text{Lb}\times \text{APb}\times \text{Tb}\times \frac{\pi }{6}$, and the form of the cervix to be an elliptical cylinder using the volume formula: $\text{Lc}\times \frac{1}{2}\text{APc}\times \frac{1}{2}\text{Tc}\times \pi$. The volumes of the uterine body and of the cervix were summed. These formulas were chosen to obtain the most reliable UV with 2D US measurements. In addition, the fundocervical antero-posterior ratio (FCR) was calculated using the formula $\frac{\text{APb}}{\text{APc}}$.

The UV and FCR were calculated in TS patients before the beginning of pubertal induction, after 6-12 months of ERT and after ≥; 36 months of ERT or ≥; 12 months after menarche.

The statistical calculations were performed using the Statistica 12 program from StatSoft and PQStat from PQStat Software. Data are presented as the median and standard deviation (SD). The α = 0.05 was assumed as the significance level. The result was considered statistically significant when p < α. Continuous variables are shown using the mean ± SD. The number and % are given for categorical variables. The normality of the distribution of variables was checked using the Shapiro-Wilk test. To compare the variables, Student’s t-test for unrelated samples was used if the distribution of the variable was consistent with the normal distribution and the variances were equal, or the Mann-Whitney test was used in the case of incompatibility with a normal distribution. Repeated measures ANOVA or Friedman’s test with Dunn’s multiple-comparison test was calculated to investigate changes in the analysed parameters over time. To investigate the relationship between continuous variables, in the case of both of them being consistent with the normal distribution, the Pearson r correlation coefficient was calculated. In contrast, the Spearman RS rank correlation coefficient was calculated when the distribution was not consistent with the normal distribution. Multiple regression was used to identify prognostic parameters for uterine development.

## Results

In 21 (52.5%) patients, the 45, X karyotype was detected, and the remaining patients had different mosaic karyotypes. The average age of estrogen induction was 12.9 ± 1.2 years and at the last control visit, at which the uterus was considered mature, it was 16.1 ± 1.6 years. The average bone age at estrogen induction was 11.7 ± 1.0 years. In 33 (82.5%) girls, menarche appeared at the average age of 15.4 ± 1.4 years. The 7 TS girls were pre-menarchal at the time of the last control visit. The first menstrual bleeding occurred after 2.3 ± 1.1 years of ERT. The characteristics of the study group are presented in **Table 1**.

**Table 1:** Clinical characteristics of the study group.

Number of patients	N = 40
Age at initiation of E2 therapy, years (range)	12.9 ± 1.2 (10.5-15.4)
Bone age at initiation of E2 therapy, years (range)	11.7 ± 1.0 (9-14)
Age at the last visit, years (range)	16.1 ± 1.6 (13.8-18.0)
Height at the last visit, cm	153.8 ± 6.5
Height SD at last visit	-1.8 ± 1.1
BMI at the last visit, kg/m^2^	23.3 ± 4.0
BMI centile at the last visit, kg/m^2^	72.5 ± 24.0
Body surface at the last visit, m^2^	1.5 ± 0,2
Growth hormone therapy, *n* (%)	39/40 (97.5%)
Karyotype 45, X, *n* (%)	21/40 (52.5%)

At the beginning of ERT, the Tanner scale was estimated as stage 1 in 34 patients (85%), stage 2 in 4 patients (10%) and stage 3 in 2 patients (5%). After 6-12 months of estrogen substitution, thelarche was as follows: stage 1 in 2 (5%), stage 2 in 18 (45%), stage 3 in 15 (37.5%) and stage 4 in 5 (12.5%) patients. At the last control visit, thelarche advancement was as follows: stage 2 in 3 (7.5%), stage 3 in 6 (15%), stage 4 in 15 (37.5%) and stage 5 in 16 (40%) patients.

**Figure 2:**
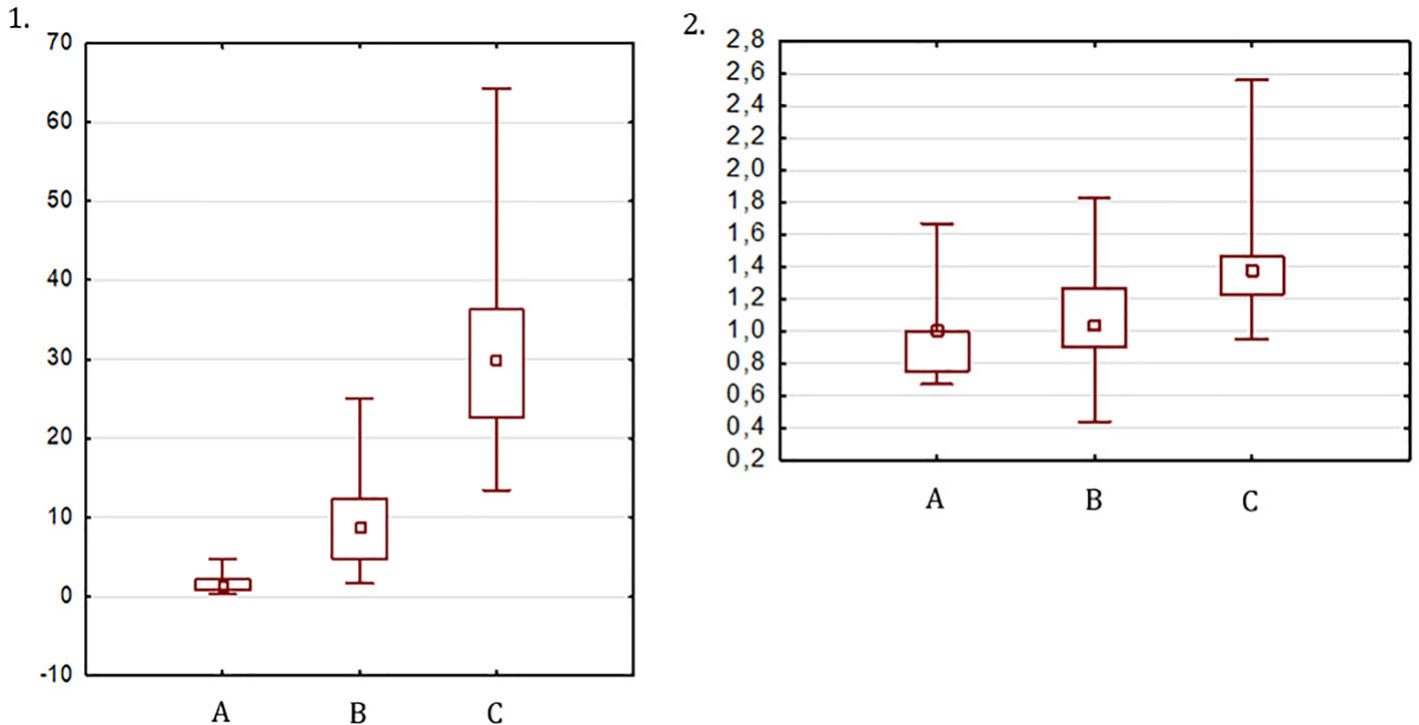
UV **(1)** and FCR **(2)**: (A) at the start of ERT, (B) after 6-12 months of ERT, (C) after 36 months of ERT or 12 months after menarche.

The UV in TS patients at the beginning of ERT was 1.55 ± 1.22 cm^3^ and was not significantly different from the UV in the prepubertal controls (1.96 ± 0.94 cm^3^, p=0.09), who were significantly younger (6.7 ± 1.0 years *vs.* 12.9 ± 1.2 years, p < 0.001). After 6-12 months of ERT, the UV increased significantly to 9.27 ± 5.63 cm^3^ (p=0.02). The volume of the mature uterus (after ≥ 36 months of ERT or ≥ 12 months after menarche) in patients with TS was 31.04 ± 11.78 cm^3^ and was significantly smaller than the UV of the postpubertal controls (45.68 ± 12.51 cm^3^, p<0.001) at a similar age (p=0.7) (**Figure 2**).

The FCR at the start of ERT, after 6-12 months and at a mature state, was 0.96 ± 0.25, 1.07 ± 0.31, and 1.4 ± 0.33, respectively (**Figure 2**). The FCR in the prepubertal and pubertal control groups was 1.04 ± 0.24 and 1.48 ± 0.27, respectively. The FCR in girls with TS at the start of ERT and at the final visit did not differ significantly from that in the prepubertal and postpubertal control groups, respectively.

The ultrasound uterine parameters are summarized in **Table 2**.

**Table 2:** Ultrasound parameters of the uterus in Turner syndrome patients and in the control group.

	TS at the initiation of ERT	Prepubertal control group	TS after 6-12 months of ERT	TS mature uterus	Pubertal control group
UV [cm^3^]					
Mean ± SD	1.55 ± 1.22	1.96 ± 0.94	9.27 ± 5.63	31.04 ± 11.78	45.68 ± 12.51
Median	1.11	1.78	8.65	29.87	43.25
Range	0.33-4.76	0.93-4.7	1.65-25.05	13.42-64.29	27.5-74.5
p-value	0.09			< 0.001	
FCR					
Mean ± SD	0,96 ± 1.22	1.04 ± 0.24	9.27 ± 5.63	1.4 ± 0.33	1.47 ± 0.27
Median	1.0	1.0	1.04	1.37	1.45
Range	0.67-1.67	0.66-1.8	1.44-1.83	0.95-2.56	0.93-2.06
p-value	0.22			0.18	

The 5th percentile for UV in the prepubertal control group was 0.94 cm^3^, which means that 38.9% of girls with TS before pubertal induction presented UVs below the 5th percentile compared to healthy prepubertal girls. The 5th percentile for UV for the control group after menarche was 27.85 cm^3^, and 39.3% of patients with TS at the last visit had UVs below this value.

The karyotype type (45, X *vs.* others) was not relevant for the UV and FCR in the pre-treatment period, after 6-12 months of therapy, or at the end of follow-up. There was a significant correlation between UV and both bone age (r=0.7, p=0.001) and serum estradiol concentration (r=0.5, p=0.02) at the start of the ERT. The UV after 6-12 months of ERT and at the final visit was not influenced by the age of ERT induction, bone age, FSH, LH, or estradiol concentration at the starting point. Moreover, the final UV was not correlated with the estrogen dose at the last consultation, the time between the estrogen induction and menarche, menarche age, BMI percentile, or body surface area. Total cholesterol and triglycerides did not change significantly during estrogen treatment.

Bone age, lipids, and hormonal parameters (FSH, LH, estradiol) before and during ERT are presented in **Supplemental Table 1**.

We considered prognostic factors for the final UV, including the chronological age, bone age, FSH concertation and UV at ERT initiation, change in UV between the initial visit and after 6/12 months of therapy, but these factors did not appear to be significant predictors.

## Discussion

Our study evaluated uterine development during pubertal induction in a homogenous group of girls with TS with hypergonadotropic hypogonadism. Most of the patients (87.5%) used transdermal estrogen therapy. The studied group was compared to the healthy population at prepubertal and pubertal ages. The mean age of TS patients at ERT initiation was more advanced than that of the prepubertal control group. The time of puberty induction (12.9 years) was in line with the recommendations (3), and the control group had to be younger, as puberty in healthy girls starts at an average age of 10.5 years (17). Most studies estimating uterine development during ERT in individuals with hypogonadism have analysed the effects of oral estrogens (13, 18–22) and/or late-onset pubertal induction (2, 12, 19); some present patients with different hypogonadism aetiologies (4) or do not compare the studied group to the control group (2, 12, 19).

The optimal imaging technique and the most objective and useful measurements of the uterus for monitoring uterine development have not yet been determined. The criteria comprising uterine maturity vary (FCR, uterine length, and UV). In our opinion, the appreciation of uterine development based on the entire size is more reliable than approaches based on a single measurement.

The research conducted by Hagen et al. (23) in healthy girls showed a strong correlation between the UV calculated with the ellipsoid formula from the 2D TAUS measurements (length, depth and width) and the volume assessed using both the three-dimensional (3D) TAUS model and the ellipsoid MRI model; however, the differences between the methods were significant and increased with increasing volume. Considering the shape of the uterus, we decided to use other geometric formulas (elliptical cylinder for the prepubertal uterus and combined ellipsoid and elliptical cylinder for the pubertal and mature uterus) to obtain the most reliable UV with 2D TAUS. Further studies with 3D US are necessary to verify this approach. Nevertheless, in contrast to 3D US, 2D TAUS is widely available in clinical practice and therefore is the standard imaging modality used to evaluate internal genital organs in girls. A validated normative model of UV, elaborated by Kelsey et al. (24), shows that the predicted volume of the uterus in healthy young women at the age of 16 years is 48.2 cm^3^ (68% prediction limit: 27.7-77.83 cm^3^), which is comparable to that in our postmenarcheal controls. To derive the model, the authors used the results of pelvic MRI from 87 paediatric patients and the results of uterine US from 1331 females aged 0-40 years. In MRI, the UV was obtained from the region of interest (ROI) drawn, whereas in US, UV was calculated using the modified prolate ellipsoid formula (24).

The lack of differences in uterine size between girls with TS prior to the beginning of pubertal induction and healthy prepubertal controls aged 5-8 years suggests that the very low estrogen secretion, which is observed in healthy ovaries in the prepubertal years (25), exerts a rather insignificant effect on uterine development. This result suggests that promoting uterine development with very-low-dose estrogen in the prepubertal years is unfounded. Trials using ultralow-dose oral ethinylestradiol as a growth-promoting agent during the prepubertal period combined with GH demonstrated a modest increase in adult height, normalization of the timing of thelarche for approximately one-quarter of the girls, and modest improvements in cognition and memory (25–28). However, as the dosing and administration of childhood estrogen have not been optimized and long-term safety has not been assessed, the addition of very-low-dose estrogen replacement in the prepubertal years in TS patients is currently not recommended (3).

In healthy girls, a significant increase in the size of the uterus is concurrent with pubertal development, and the maximum velocity of the increase in UV occurs less than a year after peak height velocity (23, 24). Pubertal induction in our TS patients resulted in a significant increase in UV, which was already evident after the first 6-12 months of ERT with low E2 doses. The fundal-cervical ratio, the other parameter evaluating uterine growth, did not change significantly during this first period of treatment. The discrepancy between these two uterine size parameters was also noticed at the final stage of uterine development: the last evaluated UV in girls with TS was significantly smaller than that in the age-matched controls, in contrast to the FCRs, which were similar in both groups. In the majority of subjects, uterine assessment was performed when the girls had a full urinary bladder. In TAUS, the full urinary bladder creates an acoustic window that facilitates evaluation of the uterus; however, it may also flatten the uterus and modify the measurements, especially when the filling is excessive. For this reason, we consider UV, the calculation of which involves all uterine dimensions (longitudinal, AP and transverse of the uterine body and the cervix) to be the more reliable parameter of uterine development and size. Because in our study, the uterus appeared sensitive to very low estrogen levels, we suggest that proper uterine measurements might be used to titrate estradiol dosages during the induction of puberty. In contrast to serum E2 concentrations, uterine growth also reflects tissue sensitivity to estrogens.

In the healthy population up to the age of 40, approximately 84% of the variation in UV is due to age alone, with a dramatic increase in uterine size observed from age 10 to 15 (24). In a study published by Burt et al. (4), which involved women with hypogonadism who received exogenous estrogens for pubertal induction from the mean age of 15 years, 84% of patients on full estrogen replacement attained FCR>1, and 48% had UVs at less than the 5th percentile of the reference group. Compared to the participants in the aforementioned study, our patients started ERT earlier (mean age 12.9 years), and although they were younger at the final evaluation (mean age 16.1 versus 24 years), they achieved better uterine development: all but two (95%) attained a mature uterine configuration with FCR>1, and 61% had a final UV above the 5th percentile of the reference group. In a study by Gawlik et al. (2), which involved girls with TS with late-onset ERT (mean age 15.1 years), the initial UV was comparable with our results, but the final UV seemed to be significantly smaller than that in our subjects (10 *vs* 30 cm^3^). Considering the postulated existence of an optimal time window for uterine development in adolescence, we can assume that in our patients, we probably applied ERT closer to this optimal window; nevertheless, we found no correlation between age at the onset of ERT and final uterine size. Karyotype, body size parameters and final estradiol dose also had no impact on the final UV.

It must also be emphasized that the smaller UV of the TS patients after puberty induction compared to the healthy population may result from a shorter period of estrogen exposure. Girls with TS usually started ERT later than natural puberty and at the final visit, although the chronological ages of the TS and control groups were comparable, their gynaecological ages were different. The average age at menarche in the Polish population is 12.6 years, similar to the mean age at the onset of ERT in our study group. Uterine growth and maturation may continue beyond the time of menarche; therefore, patients with TS should be monitored for further development of the uterus.

There are several limitations of our study. The first one is study design (retrospective analysis). The second limitation, which was already mentioned, is the imaging technique used to evaluate UV. Further studies with the use of 3D US, which facilitates the acquisition of volumes that are more reliable and less dependent on the examiner, could provide more accurate data. The third limitation is the size of the study group and of the control groups and the relatively short time of observation. As the maturation of the uterus continues beyond the first menstrual bleeding, extension of this study and evaluation of the uterus in patients at the childhood-adulthood transition should be performed. Finally, we cannot exclude periodic non-compliance with ERT in some of our girls with TS, which could have affected the results.

## Conclusions

Prior to the onset of ERT, teenage TS patients with gonadal dysgenesis had uterus similar in size to the uterus in late prepubertal healthy girls. Pubertal induction in TS patients results in a significant increase in UV, which is detectable after 6 to 12 months of ERT with the smallest recommended doses of E2; therefore, UV measurements may be useful in monitoring ERT. The mature uterus in adolescents with TS is smaller than that in the age-matched healthy population; however, most girls with TS achieve UVs above the lower normal range of those in healthy girls. The factors that determine this difference remain to be elucidated.

## Data Availability Statement

The original contributions presented in the study are included in the article/**Supplementary Material**. Further inquiries can be directed to the corresponding author.

## Ethics Statement

Ethical review and approval was not required for the study on human participants in accordance with the local legislation and institutional requirements. Written informed consent to participate in this study was provided by the participants’ legal guardian/next of kin.

## Author Contributions

MO-M: substantial contributions to conception and design, acquisition of data, analysis, and interpretation of data, drafting the article, final approval of the version to be published. LD: analysis and interpretation of data, drafting the article, final approval of the version to be published. BR-P: acquisition of data, revising the article critically for important intellectual content, final approval of the version to be published. MN: substantial contributions to conception and design, drafting the article and revising it critically for important intellectual content, final approval of the version to be published. KK: substantial contributions to conception and design, acquisition of data, analysis, and interpretation of data drafting the article and revising it critically for important intellectual content, final approval of the version to be published. All authors contributed to the article and approved the submitted version.

## Conflict of Interest

The authors declare that the research was conducted in the absence of any commercial or financial relationships that could be construed as a potential conflict of interest.
