# Supplementary material for: Uterine Development During Induced Puberty in Girls with Turner Syndrome
Source: Front Endocrinol (Lausanne). 2021 Jul 6;12:707031. doi: 10.3389/fendo.2021.707031 (PMC8290974; doi:10.3389/fendo.2021.707031)
Supplement: Supplementary file 1 [file Table_1.docx]

Supplementary Material

## Supplementary Tables

**Supplemental Table 1.** Bone age, serum concentrations of lipids and hormonal parameters before and during E2 therapy in the TS group.

|  | At the initiation of E2 therapy | After 1 year  of E2 therapy | After 2 years  of E2 therapy | After 3 years  of E2 therapy |
| --- | --- | --- | --- | --- |
| Mean bone age [years]  Range | 11.8  9-14 | 12.4  10-14.5 | 13.3  12-15 | 13.5  12.5-14.5 |
| Mean FSH [mIU/ml]  Range | 87.5  24-150 | 37.4  2.5-80.8 | 40.6  0.4-81.5 | 22.9  0.3-64.2 |
| Mean LH [mIU/ml]  Range | 18.6  0.9-40.8 | 11.2  0.0-27.3 | 14.2  1.3-43 | 10.0  0.2-30 |
| Mean estradiol [pg/ml]  Range | 14.8  10-31 | 24.8  10-70 | 30.7  10-92 | 57.1  16-160 |
| Mean total cholesterol [mg/dl]  Range | 189.1  129-330 | 184.1  125-339 | 183.0  139-258 | 177.5  93-251 |
| Mean triglycerides [mg/dl]  Range | 114.1  43-319 | 106.0  48-213 | 107.0  44-267 | 84.2  51-158 |
